# Photobiomodulation: A Promising Safe Therapy for Alzheimer's Disease

**DOI:** 10.1002/alz70859_100584

**Published:** 2025-12-25

**Authors:** Guillaume Blivet, Hugo Cavadore, Marwan N. Sabbagh, Jacques Touchon

**Affiliations:** ^1^ REGEnLIFE, Montpellier France; ^2^ University of Montpellier, Montpellier France; ^3^ Barrow Neurological Institute, Phoenix, AZ USA; ^4^ CHU Montpellier, Montpellier France

## Abstract

**Background:**

The pathophysiology of Alzheimer’s Disease (AD), is characterized by a double proteinopathy leading to oxidative stress, neuroinflammation, cerebral lesions, and dysbiosis of the brain‐gut axis microbiota. Photobiomodulation (PBM), a non‐invasive therapeutic approach utilizing low‐level red or near‐infrared light, can be applied transcranially as a potential treatment for AD. Preclinical studies have demonstrated that PBM reduces oxidative stress and inflammation, enhances cerebral blood flow, and promotes neurogenesis and synaptogenesis, addressing key pathological mechanisms of the disease. Given the growing interest in PBM as an innovative intervention, a comprehensive evaluation of current clinical studies on PBM in AD is both timely and essential. This analysis critically examines the existing evidence on PBM therapy for AD, assessing its therapeutic potential and feasibility for integration into combination treatment strategies.

**Method:**

Since 2017, eight clinical studies meeting rigorous methodological criteria have been included. Of these, four were double‐blinded randomized controlled trials (RCTs) versus sham treatments, one was a single‐blinded RCT versus sham, and three were open RCTs. These trials investigated PBM applications across various patient populations, including those with AD (n=3), AD or dementia (n=2), dementia (n=2), and Mild Cognitive Impairment (MCI) (n=1).

**Result:**

Clinical studies consistently demonstrate the safety of PBM in AD and related dementias. Preliminary evidence also indicates potential cognitive benefits. However, the evidence remains limited due to small sample sizes, PBM technologies and methodological heterogeneity. These limitations hinder the establishment of conclusive clinical recommendations. T Research on treatment parameters, such as photonic emissions and dosimetry, session duration and treatment period, would be valuable to optimize the therapeutic benefit. Double‐blinded randomized controlled trials (RCTs) with adequate sample sizes are necessary to conclusively demonstrate PBM’s therapeutic efficacy.

**Conclusion:**

PBM therapy shows considerable promise as a safe, non‐invasive treatment for AD. However, its widespread clinical application depends on rigorous validation of efficacy through high‐quality studies and the development of standardized treatment protocols. The next phase of research will be instrumental in determining whether PBM can transition from an experimental approach to a mainstream therapeutic option for AD. Ongoing and forthcoming clinical studies are expected to contribute to this body of evidence.

